# Good behavior game – study protocol for a randomized controlled trial of a preventive behavior management program in a Swedish school context

**DOI:** 10.3389/fpsyt.2023.1256714

**Published:** 2023-10-05

**Authors:** Dariush Djamnezhad, Martin Bergström, Per Andrén, Björn Hofvander

**Affiliations:** ^1^Department of Clinical Sciences Lund, Lund Clinical Research on Externalizing and Developmental Psychopathology, Lund University, Lund, Sweden; ^2^Administration of Compulsory Education Department, Malmö, Sweden; ^3^School of Social Work, Lund University, Lund, Sweden; ^4^Department of Clinical Sciences Lund, Lund University, Lund, Sweden; ^5^Child and Adolescent Psychiatry Skåne, Lund, Sweden; ^6^Department of Forensic Psychiatry, Region Skåne, Sweden; ^7^Department of Psychiatry and Neurochemistry, Centre of Ethics, Law and Mental Health, University of Gothenburg, Gothenburg, Sweden

**Keywords:** good behavior game, study protocol, behavior management, randomized controlled trial, school-based intervention, prevention, conduct problems, pragmatic trial

## Abstract

**Background:**

Early conduct problems and school failure are prominent risk factors for several adverse outcomes in later life. With the potential of reaching many children at early stages of their life, school-based interventions constitute a valuable approach to universal prevention. Good behavior game (GBG) is a promising school-based behavior management program, having shown immediate reductions in conduct problems along with several long-term positive effects. Adapting interventions to new contexts may however affect their effectiveness. The current study aims to evaluate the effectiveness of a Swedish adaption of GBG under pragmatic conditions. The intervention is hypothesized to reduce conduct problems in the classroom (primary outcome). Secondary analyses will investigate changes in conduct problems in common school areas, classroom climate, teacher collective efficacy, on-task behavior, as well as investigating behavioral management practices, implementation, and barriers to implementation.

**Methods:**

This is a cluster-randomized trial with two parallel groups. Schools will be randomized (1,1, stratified by their areas sociodemographic index score) to be provided training in GBG or perform business-as-usual. The intervention and data collection lasts for a school year. Data will be collected at three time points: at baseline in the beginning of the school year (prior to training in GBG), after three months, and after nine months (at the end of the school year; primary endpoint). Data consists of teacher-rated measures of conduct problems, classroom climate, teacher collective efficacy, behavior management practices, and implementation factors, along with demographic factors. In addition, data will be collected by independent and blinded observers using corresponding measures in a subset of randomly chosen classrooms. Procedural fidelity will be rated and collected by GBG-trainers during nine observations throughout the school year. Statistical analysis will include frequentist intention-to-treat analysis, and comparisons of estimates with a corresponding Bayesian model using weakly informative priors. The study has currently completed data collection.

**Discussion:**

This study will provide knowledge in universal prevention and school-based interventions with high reach, as well as specific knowledge concerning the effectiveness of an adapted version of GBG under real-world conditions, along with factors affecting its implementation and effects.

**Clinical trial registration:**

ClinicalTrials.gov, identifier NCT05794893.

## 1. Introduction

Preventive interventions in schools have the potential to teach skills and create nurturing environments for many children over a long course of time. School-based interventions can yield positive effects on social and emotional skills, mental health, externalizing behavior, and academic skills (1). Due to their potentially high reach, these interventions can include many children in a cost-effective way (2). Furthermore, these interventions can be launched and delivered before the onset of students’ difficulties, e.g., conduct problems (3). One such established approach to school-based universal prevention is good behavior game (GBG) (4). GBG is a behavioral management program for younger school children that utilizes game-like features, such as teams, objectives, and a scoring system, in order to foster skills that are conducive to self-regulation and task-oriented classroom environments. The intervention is manualized and integrated by teachers in their regular teaching activities.

Previous studies have shown that GBG reduces children’s conduct problems in comparison to control interventions (5). Conduct problems as defined here are patterns of behaviors in which the basic rights of others or major age-appropriate societal norms or rules are violated, such as aggression (6). The prevalence of conduct problems varies considerably depending on study and sample, but even early-onset or life-course persistent conduct problems, the classifications associated with highest risk and severity, are reported to include 8%–25% of all children (7–10). Barring the most direct consequences for the individual and immediate environment, conduct problems increase the risk for symptoms related to anxiety and depression in childhood (11). These internalized symptoms, along with conduct problems, are linked to school failure (12, 13). Long-term, the presence of conduct problems in childhood increases the odds of mental health issues, aggression, criminality, higher consumption of alcohol and cannabis, poorer general health outcomes, as well as poorer educational and occupational outcomes in adulthood compared to individuals with low levels of conduct problems in childhood (14, 15). Following this logic, reducing conduct problems in childhood should reduce the risk of multiple adverse outcomes in a longitudinal perspective. Consequently, early prevention such as GBG has demonstrated positive effects all the way to young adulthood, including reductions in suicide ideation, substance use, and violent and criminal behavior, compared to control interventions (16–19). Taken together, research points to GBG as a promising preventive intervention across diverse student populations (20).

Despite earlier demonstrations of efficacy in one setting, positive effects are not guaranteed when transferring to new settings. For example, GBG was evaluated in a randomized controlled trial in England with no immediate main or subgroup effects (21). One explanation could be that a new setting needs a specific adaptation in order to retain efficacy (22). Likely attributable to multiple factors (23), it is not uncommon for interventions to lose efficacy when attempting replication outside the original site (24). Implementation is likely also a critical factor for success (25, 26), which may be influenced by contextual factors when implementing GBG and similar interventions (27). Implementation may be further compromised in a natural, or non-research, setting as adaptations are made reactively, e.g., due to lack of time and resources (28). Taken together, there is an apparent need to evaluate GBG under local and naturalistic circumstances. As such, the intention for this study is to conduct a pragmatic type of trial under local real-world conditions, rather than an explanatory study under ideal circumstances (29). In this case, the intention is to evaluate GBG when it has been entirely funded, chosen, translated, adapted, and implemented by a practically oriented organization and infrastructure, with the research group entering later in the process and attempting to minimize its influence. Consequently, this is the first randomized controlled trial of GBG with an explicit focus on being a pragmatic trial. See the PRECIS-2 table provided in Supplementary Table S1 for additional details regarding the trial’s pragmatic orientation.

The primary objective is to evaluate the effects of GBG-training on decreasing conduct problems in elementary school classrooms as compared to schools conducting business-as-usual (BAU). The secondary objectives are to evaluate the effects of GBG-training on conduct problems in common school areas, classroom climate, collective teacher efficacy, behavioral management practices and on-task behavior. With GBG compared to BAU, teacher-rated conduct problems in the classroom are expected to decrease at both 3- and 9-month follow-up. Conduct problems in common school areas are only expected to decrease at 9-months follow-up since relevant generalization components in GBG are implemented after the 3-month follow-up. It is also hypothesized that observer-rated measures will corroborate corresponding teacher-rated measures. The role of sociodemographic factors and implementation as moderators will be investigated. In addition, the role of contextual factors as possible barriers and facilitators for adoption and implementation will be investigated.

## 2. Methods and analysis

### 2.1. Design

The study is a parallel group, cluster-randomized controlled superiority trial. Schools will be chosen as the unit of randomization to minimize contamination effects and to follow the municipality’s chosen strategy for implementation. This chosen strategy implements GBG in all K–3 (typically corresponding to children aged 6–9) classrooms simultaneously in every included school, rather than allowing for implementation in single classrooms in different schools. The group ratio will be 1:1 and randomization will be stratified using an aggregated sociodemographic index score used by the municipality to allocate school funding.

As the study and intervention lasts for a Swedish school year (August to June the following year) randomization will be conducted in May, before the end of the preceding school year. This is to ensure that schools have a similar amount of time to prepare for GBG as corresponding to their usual practice, as well as allowing the research group time to prepare all schools for data collection procedures. Randomization will be conducted and implemented by the research team. After recruitment all schools will be stratified as higher or lower than the city average on the stratification variable, i.e., the municipality’s sociodemographic index score, which has a centered mean at 100. Following this procedure, schools will be assigned a computer-generated number between 0 and 1 using the statistical software SPSS (RRID:SCR_002865), where schools with the highest numbers in each stratum will be placed in the intervention group. The research group will then notify schools of their study allocation, which will either be the intervention group who will receive training in GBG, or the control group, who will be conducting BAU. Schools in the control arm will be offered GBG-training in the following school year as an incentive to participate. GBG will be conducted by teachers who receive training during the study from certified GBG-trainers provided by the municipality.

**Figure 1:**
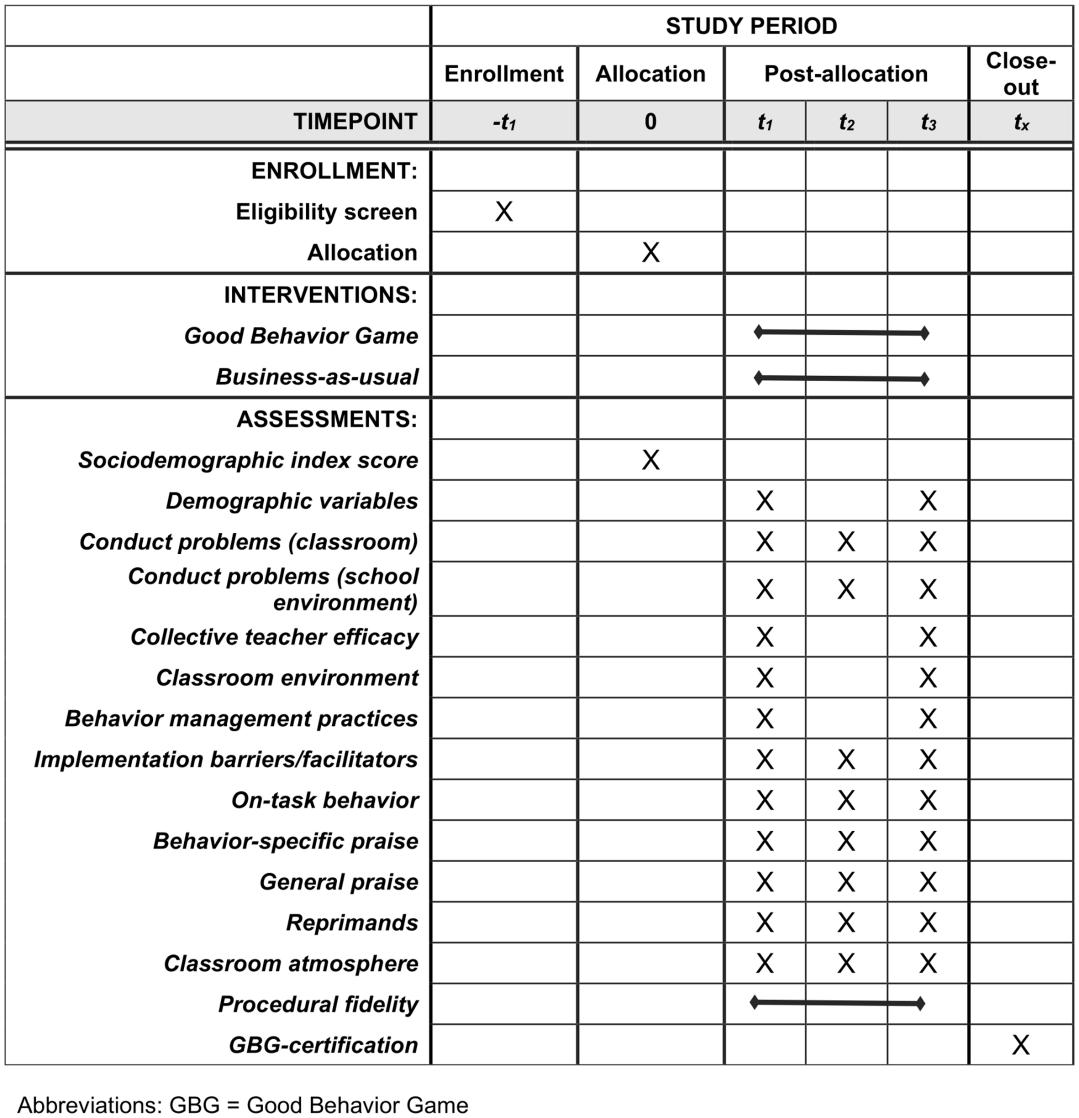
SPIRIT schedule of enrollment, interventions, and assessments.

Measurements will primarily be concentrated along three timepoints, with each data collection period being limited to three weeks: Baseline (T1), 3-month follow-up (T2), and 9-month follow-up (T3 – primary endpoint). This corresponds to training in GBG which is spaced within the duration of a Swedish school year. Baseline measurements will be conducted post-allocation, just before start of training and intervention, to ensure the inclusion of all newly enrolled students. Data is primarily collected through teacher-rated, observer-rated, and trainer-rated measurements, though trainer-rated measures are collected continuously and not constricted to the 3-week measurement periods. A list of measures and their sequence are summarized in the SPIRIT-figure (Figure 1). All measures are collected on a classroom level. The study will be set at Sweden’s third largest municipality (Malmö), who is currently piloting the preventive framework Communities That Care (CTC) (30). See the trial registration on ClinicalTrials.gov (NCT05794893) for more details on study sites.

All included schools will be assigned a site coordinator. The site coordinator acts as a liaison and facilitator for the trial coordinator (DD) for all matters concerning the study. The roles are primarily for facilitating data collection through teacher forms and independent observers. GBG-trainers are expected to have independent contact with schools regarding implementation but may also have contact with the trial coordinator. The research group will encourage compliance to data collection procedures under all circumstances.

### 2.2. Selection of subjects

To follow usual practice for the intervention, eligible schools are those with students in grades K–3. Exclusion criteria consist of schools where classrooms are already implementing GBG or schools who primarily have special education classrooms. However, inclusive classrooms using the standard Swedish curriculum with a few students receiving special education may still be included.

Recruitment will also follow usual conditions for the municipality, which in this case means that recruitment will be headed by trainers and facilitators working with CTC. The intervention and consequent participation in the study will first be offered to eligible schools in pilot areas for CTC. Schools with matching risk and protective factors according to CTC will be prioritized. Due to GBG already being implemented in several pilot areas, only a few schools are expected to sign up at this phase. As such, recruitment will be broadened in a second stage where GBG and participation in the study is offered to all remaining eligible schools in the municipality. Interested schools will then be jointly assessed for eligibility by the municipality and the research group. In the case of unsuccessful recruitment, other geographically close municipalities could be included in the recruitment phase.

In order to maximize statistical power for this sample, the number of recruited schools need to remain at the municipality’s maximum capacity for simultaneously implementing GBG, which is roughly 3–5 new schools at the time of the study for the intervention group. This corresponds to approximately 450–750 students based on typical school and class sizes in the area. It is important to note that this sample size was judged as acceptable with an earlier design and power analysis for this study that included individual level variation. However, this power analysis has not been updated for the current design that measures primarily on a classroom level, which is likely to yield lower statistical power, even with the intervention group at full capacity.

### 2.3. Interventional methods

In 2015, the Swedish municipality of Malmö launched a process of exploring and adapting GBG to a Swedish context for use within a general preventive framework (CTC). While GBG is American in origin, several versions have been studied both domestically and internationally since its inception (31, 32). To ensure the closest fit, the municipality opted for a version adapted and studied in a Dutch-speaking context (33–35). There are some notable adaptations in the Dutch version of GBG compared to the contemporary American version (from American Institutes for Research) it was based on, such as students encouraging each other in behaving appropriately, and teachers not mentioning student’s rule violations (34). When developing the Swedish version, the intention was to keep adaptations on a surface level, rather than changing deep structure. As such, only a few adaptations were made in adapting the Dutch version to a Swedish context, besides translation. Most notably, rewards are less focused on tangibles and more on desired activities compared to the Dutch version. Some minor changes were also made to increase fit to the Swedish school year. The complete Swedish adaptation (named “Höjaspelet”) has been tested in a pilot study, but without a control group and with high attrition (36).

GBG (Höjaspelet) is a teacher-driven and manual-based behavioral management program for the classroom. It is played out like a game using interdependent groups. The game is integrated with regular classroom teaching. Based on teacher observation and assessments of student’s shyness and rule infracting behavior, students are divided into balanced teams. Each team customizes their name and poster to be used for the game. Each session the teams receive a set of cards representing points. The game starts with three rules, which are formulated as positively stated and observable behaviors (e.g., “Raise your hand to speak,” “Work independently” or “Share with your classmates”) and paired with visual aids. During the game, the teacher frequently delivers behavior-specific praise contingent on students or groups following the rules. Rule infractions prompt the teacher to remove a point card from the team discreetly, but otherwise ignoring the infraction rather than reprimanding. Teachers are encouraged to maintain at least a 4:1 praise-to-reprimand ratio. After the game, all teams that still have at least one card are included in a reward. Teachers are encouraged to ensure that all teams can and should win, e.g., by increasing the number of point cards available. Wins are tallied on each team’s poster.

The intervention is divided into an introductory phase, expansion phase, and generalization phase. The phases are ordered sequentially and last about 3 months each, with the introduction phase being somewhat longer and the generalization phase being somewhat shorter. The class is divided into new teams prior to each phase. The function of the different phases is to gradually generalize new behaviors outside the game context. This is generally done by increasing game time and fading out components of the game (e.g., fewer, and more delayed rewards). Game time is set to gradually increase during the introduction phase, from 10 min to about 45 min. With the help of transition supports and breaks, the game time may increase to as much as 3 h during the expansion phase.

Game time does not necessarily increase during the generalization phase, instead focus is placed on incorporating components of GBG outside the specified game time. For example, the generalization component of this phase may entail teachers still referring to three rules and delivering praise contingent on adhering to the rules, while maintaining a 4:1 praise-to-reprimand ratio. At the same time, the teacher should remove the usage of teams, their respective posters, and their point cards. At first, this method of generalization is only used for about 5 min immediately following a regular game session. As the phase progresses, it is conducted during longer periods of time, and not necessarily as a direct extension of a regular game session. Teachers are also prompted to have students discuss and share reflections regarding the game once in a while during this phase.

The number of game sessions is set to 3 times a week during all phases. This default format is designed to last for a school year with a class that is naïve to GBG, which is the focus of this study. Teachers may start the school year in a later phase when conducting GBG with students who already have experience with GBG.

Although this Swedish adaptation keeps GBG within the classroom environment, the municipality uses a school-wide implementation process, meaning all K–3 teachers within a school are required to receive training and implement the intervention simultaneously. Teachers receive about 3.5 h of group instruction prior to every phase. The trainers also observe the teachers approximately once per month (nine times in total during the school year), using a standardized form, and delivering feedback to the teacher after every observation.

All training in GBG is conducted by certified trainers in GBG employed by the municipality. The standard path to becoming certified as a GBG-trainer is by first becoming certified in conducting GBG in the classroom. After this, a person can be certified as an internal GBG-coach by observing and giving feedback on procedural fidelity at least twice in all three phases, accompanied by a certified trainer. The final step is to conduct training sessions together with certified trainers during a year of implementation before becoming a certified GBG-trainer, making the entire process approximately three years long.

The trainers in this trial will be a mix of trainers who have either been certified through the standard path by Swedish trainers or certified as the first generation by Dutch trainers which did not include the first certification, i.e., implementing GBG with fidelity in a classroom as a teacher. To become an independent site that could certify new teachers and trainers, the first generation of Swedish trainers were trained in the Dutch version of GBG by the CED Group Foundation (a non-profit organization).

As the study is designed to test the effectiveness of GBG under naturalistic conditions, BAU was chosen as a comparator as the objective is to compare GBG to existing behavioral management strategies that schools already have in place or will naturally implement.

Since the study follows naturalistic conditions, both in how the intervention is implemented, and how schools proceed without the intervention, the research group does not attempt to control teacher practice, e.g., implementing measures to increase fidelity or control concomitant care. No criteria are set for discontinuing or modifying any allocation from the research group. Teachers in the intervention group can modify or be noncompliant regarding the intervention, and teachers in the control group can attempt to use practices taught in GBG without training. For this reason, behavior management practices will be tracked in both arms. It will primarily be up to schools and training staff to ensure implementation in the experimental arm. The research group will not directly support implementation efforts in this study.

### 2.4. Measures

#### 2.4.1. Teacher-rated measures

One teacher from each included class will respond to a demographic questionnaire developed for the trial concerning the number of students in each class, number of boys (to assess gender distribution), number of students where Swedish is a secondary language, number of students (partially or wholly) excluded from ordinary classroom activities, additional staff in the classroom, and experience with the current class. Demographic items are assessed at T1 and T3. The intervention group receives two additional items at T3 regarding the number of GBG-sessions they had the previous week and if they expect that they will continue using GBG during the next school year.

##### 2.4.1.1. Conduct problems

The primary outcome for this study is conduct problems in the classroom. This will be measured using the scale “Problem Behavior in the Classroom Last Week,” listing 20 items concerning conduct problems commonly found in school classrooms, e.g., verbal assault directed towards other students, disturbing or distracting other students, and physically assaulting other students. A secondary outcome is conduct problems in school environments, which will be measured using the scale “Problem Behavior in the School Environment Last Week.” The scale remains similar to its classroom counterpart and consists of 15 items concerning conduct problems experienced in school common areas. For both scales, the teacher bases their ratings on the week prior to assessment and rates all items on a scale from 0 (no times last week) to 4 (several times a day). Both scales were translated from Norwegian to Swedish and have shown sound psychometric properties in previous Norwegian studies, with internal consistency at α = 0.88 and α = 0.84 for the respective classroom and school environment scales (37–39). Conduct problems will be assessed at all three time points.

##### 2.4.1.2. Teacher collective efficacy

Teachers will rate 12 items on a collective efficacy scale. The scale concerns the perceived efficacy of teachers in the school, as a collective, being able to teach all students attending the school or if they experience considerable barriers. It revolves around factors such as teachers not being able to motivate students, students’ sociodemographic factors being too difficult to overcome, or that teachers in the school do not possess the necessary skills to deal with problem behaviors. Items are rated on a 0–5 Likert scale and were translated to Swedish from a Norwegian version of the scale (38, 40). Internal consistency ranged from α = 0.82 to 0.85 in a Norwegian sample (41). The scale will be assessed at T1 and T3.

##### 2.4.1.3. Classroom environment

To assess classroom environment, teachers will rate 14 items on the Classroom Environment Scale. The scale concerns the learning climate in the classroom (e.g., cooperation between students, if students are task-oriented or if students are engaged). Items are rated on a 0–3 Likert scale. The scale has been translated from Norwegian to Swedish. Internal consistency ranged from α = 0.83 to 0.85 in the most recent Norwegian sample (39). The scale will be assessed at T1 and T3.

##### 2.4.1.4. Behavior management practices

Behavioral management practices will be assessed using 22 items that represent an assortment of behavioral management practices that may be used by teachers in the classroom (e.g., giving praise to students for desired behaviors, using harsh reprimands, and practicing social skills). The items are divided into the scales Positive Behavior Support and Behavioral Correction. Items are rated on a scale of 0–6, ranging from using a certain strategy 0 times to using it more than 20 times, all during the last 30 days. The items were developed in Norway and translated to Swedish. The Norwegian sample supported the use of the two factors Positive Behavior Support (α = 0.74 to 0.76 using nine items) and Behavioral Correction (α = 0.57 to 0.66 using eight items) (41). The instrument will be used at T1 and T3.

##### 2.4.1.5. Contextual barriers and facilitators for implementation of GBG

To assess possible contextual barriers and facilitators for implementation, along with their perceived strength as factors, teachers in the experimental group will rate the pragmatic context assessment tool (pCAT) (42). The instrument consists of 14 items regarding implementation barriers and facilitators drawn from the Consolidated Framework for Implementation Research (CFIR). Each item has two separate ratings. The first rating is used to determine if the statement is a barrier, neutral or facilitating factor. The second rating determines the strength of the factor, i.e., if the factor is considered to have a weak/no effect or strong effect. pCAT was translated and adapted to the study by the research group. It will be assessed at all three time points, although T3 will be the focal point for analysis as teachers will be more experienced with GBG and likely have a more accurate estimate of what factors are perceived to influence implementation.

#### 2.4.2. Observer-rated measures

To complement teacher-rated measures, blinded and independent observers will visit a subset of included classrooms. To reduce costs, only one classroom per grade will be observed at each time point. The observed classrooms will then be chosen at random from each grade level. If a school only has one class per grade, all classes will be observed in that school. Each of these classrooms will be observed once during the same 3-week measurement periods the teachers fill in questionnaires from T1–T3. Each observation takes approximately 30 min to conduct. Observers will be trained by attending a workshop where they practice using the measurements on video sequences of an elementary school classroom. The observers will also be given a manual with behavioral targets and definitions, along with a detailed description of all procedures.

Observers will be hired independently by the research group to not be affiliated with the municipality. To blind the observers, they will only be given general information about the study (e.g., a study about classroom climate), i.e., no information about the study’s design or there being an intervention will be provided. The schools will be given instructions to not conduct GBG-sessions during observations and to not reveal any elements of the study to the observers. While some artifacts of GBG may be visible (e.g., team posters), they should blend with other visual objects that are common in classrooms. No circumstance or procedure for unblinding has been deemed applicable, and no other blinding (e.g., for intervention providers) has been deemed possible at this point.

The observational procedure starts with observers rating on-task behavior using Planned Activity Check (PLACHECK) (43); counting the number of task-oriented students each 3-min interval. The observers simultaneously count the frequency of three different types of teacher response to student behavior: General praise (e.g., “Good job!”), behavior-specific praise (e.g., “Well done sharing those pencils!”), and behavioral corrections or reprimands (e.g., “Stop that!”). These measures are performed continuously for 20 min.

After at least 30 min in the classroom, the observer rates 10 items on a Swedish translation of the Classroom Atmosphere Measure. Using behavioral indicators, items are rated on a scale of 1–4, with the option of scoring 0 if the item is not possible to rate or not applicable. A previous study measured internal consistency at α = 0.92 and kappa coefficients ranging from 0.62 to 0.81 (44).

#### 2.4.3. Fidelity

Certified trainers in GBG observe teachers in the experimental group on nine occasions, three for every phase in GBG, during the school year. The trainers rate procedural fidelity at each observation, before, during, and after the GBG-session using a standardized form. Scores will be averaged for the observation sessions to create a score for procedural fidelity.

Due to previous difficulties, collecting data on dosage (e.g., frequency and duration of GBG-sessions) is no longer a part of the trainer’s usual practice. To ensure that there is some proxy for dosage, trainers are asked to collect data on dosage through a weekly chart that tracks whether the teacher has played GBG 3 times during the week or not. Further measures regarding dosage have not been judged as feasible.

Trainers may certify teachers at the end of the school year. The requirements for certification in GBG are participation in all three training sessions, conducting at least 60 GBG-sessions during the school year, being observed by a certified trainer at least nine times and judged to conduct sessions with enough fidelity. This will constitute a binary variable, serving as a general measure for what the trainers assess as being passable implementation quality, to complement procedural fidelity.

### 2.5. Data analysis

As this study aims to investigate the effects of introducing GBG under real-world circumstances, whether the sample is compliant to the intervention or not, data analysis of the primary and secondary outcomes will follow intention-to-treat (ITT) procedures. The primary objective can be described as the difference in change over time in conduct problems between the group receiving GBG and the group receiving BAU. This can be expressed as a general linear model where conduct problems would signify the primary outcome of interest (denoted as Y ), with intervention group, time, and stratification variable (sociodemographic index score) being converted to dummy-scores and denoted as $\beta_{1}$ , $\beta_{2}$ , and $\beta_{3}$ respectively, using an interaction term ( $\beta_{1}\times \beta_{2}$ ) for intervention group and time. This approach entails some statistical considerations. One consideration is adjusting for the standard errors being affected by clustering (45). Since the municipality only has capacity to implement GBG in a low number of schools, considerations have to be made regarding the degree of precision in statistical models with few clusters. One option is to use a Bayesian ITT-model, which could render more precise estimates compared to corresponding frequentist models as long as default naïve priors aren’t used (46). Data for priors may be limited, particularly for some of the secondary outcomes, in which case weakly informed priors will be used. The estimates can also be compared with the corresponding frequentist ITT-analysis. As priors can have notable effect on estimates (47), sensitivity analyses will be conducted.

Analyses for secondary outcomes, including observational measures, will in large follow similar procedures, i.e., as outcomes in linear univariate models. Statistical analyses related to implementation will be treated differently. To assess implementation as a moderator, a parameter for procedural fidelity will be added to the statistical model for the primary analysis. Note that this is contingent on the chosen proxy for implementation to have some degree of variation. An earlier study reported high procedural fidelity across the board, with only dosage being suboptimal (21). This indicates that dosage may be a more potent moderator to investigate compared to procedural fidelity. Due to the previously mentioned difficulties in collecting data on dosage, the teacher-rated variable for number of GBG-sessions last week may be used as a proxy for dosage instead of the trainer-collected data on number of GBG-sessions for the school year.

While all data collection is handled by employees who have the same or similar formal requirements, the theoretical requirements of data “missing completely at random” are high. It is also possible that data could be “missing at random”, but in this case there are many unobserved variables that could potentially explain systematic differences between missing and observed data. Should missing data occur, it will primarily be assumed as “missing not at random”. Consequently, listwise deletion will be the default option to handle missing data. Multiple imputation will also be considered in cases where arguments can be made for data being missing at random, or where inclusion of auxiliary variables is deemed suitable. However, no measured variables in this study are currently expected to predict or correlate with potential missing data.

## 3. Discussion

The current trial will evaluate the effects of GBG compared to BAU, primarily using teacher ratings and blinded observer ratings across three time points during a school year. As previous trials have demonstrated efficacy for GBG, this trial places larger focus on effectiveness or pragmatic aims, along with testing the transportability of GBG. The pragmatic focus means that allocations mirror real-world conditions as closely as possible, with the research group trying to minimize restriction and its own impact regarding all aspects of school practice in the trial. This should add to the literature concerned with moving GBG along a translational research pipeline (48, 49). Also concerning translational research, the study should be able to partially apply findings within the RE-AIM framework (50), which focuses on the reach, effectiveness, adoption, implementation, and maintenance of public health interventions. Some key variables for assessing these areas are the number of students exposed to the intervention, number of teachers providing GBG, their level of fidelity, and whether teachers expect to continue their use of GBG. Should these parameters be favorable, even smaller effect sizes could be valuable from a public health perspective.

Smaller effect sizes may be expected as this study employs a parallel group design with observer-rated measures taking place strictly outside game sessions. Although the teacher-rated measures should take behaviors both in and outside GBG-sessions into consideration, moderate to large effect sizes for GBG are more common in single-case experimental designs using observational measures while the game is played (51). A benefit with the current design is that it allows investigation of whether behaviors generalize across time and context when implementing GBG, e.g., if conduct problems are reduced in classrooms as well as common school areas, or whether potential effects are immediate and transient, rather than sustained over the school year.

The lack of restrictions in this pragmatic trial also increases the importance of tracking behavioral management practices in the control group. Usual care or practice has a history of only being vaguely defined and controlled for (52). The efficacious behavioral management practices found in GBG could potentially be more widely disseminated among teacher staff in this sample compared to previous studies, e.g., due to generally increased knowledge or teacher mobility within the municipality. To address this, behavioral management practices will be tracked in both groups by independent observers and teacher-rated measures.

The study design poses some methodological considerations, most notably the reduction in statistical power by the limited sample size and classroom-level variables. An earlier version of the study used individual-level variables (with a corresponding three-level power analysis) in conjunction with consent forms where parents could actively opt-out their children from the data collection, similar to the design used by the English GBG-trial (53). However, the Swedish Ethical Review Authority required active consent from all parents. This prompted a change in design to remove individual-level variables as there were major concerns with attrition using active consent forms, particularly in areas with lower socio-economic status. Further, any potential changes had to be made rapidly as preparations and implementation of GBG need to be timed accordingly to the school year, and the pool of local eligible schools is expected to reduce for each implementation cycle. This also precluded the inclusion of an updated power analysis corresponding to the new design and pre-registering the study before recruitment. This limitation should be considered in light of the difficulties in achieving suitable sample sizes in highly pragmatic conditions. A potential benefit is that the current design is relatively low-cost and lightweight, making it suitable for pragmatic aims. This may encourage more trials when organizations import or develop interventions before scaling up, which could be a significant investment considering each trainer require at least three years of development in this case. Newer interventions may however still need large trials under ideal conditions to first determine efficacy.

An additional consideration is that the primary outcome is teacher-rated and not blinded. This is only partially addressed using blinded and independent observers as they only observe a subset of classrooms due to financial limitations, specifically one classroom for each grade in each school. The blinded measures will still serve an important role in corroborating teacher-rated outcomes, with discrepancies between observer-rated and teacher-rated measures potentially implying weaker effectiveness. For example, positive changes in teacher-rated measures, but not in observer-rated measures, could potentially imply rater bias or that changes in behavior have not generalized outside of game sessions. Another possible limitation is that the study assesses change in multiple outcomes, meaning multiple independent comparisons. This could potentially increase the risk of type I error but should be ameliorated by having pre-specified a primary outcome, primary endpoint, and methods for data analysis. The breadth of outcomes also allows for the evaluation of more positive goals. For example, conduct problems could in theory be suppressed by using highly aversive or restrictive methods, but such methods would likely have detrimental effects on other outcomes assessed in this study.

In conclusion, this trial should inform the literature on how an adapted version of GBG fares in a naturalistic scenario. This has implications for how preventive interventions with high reach can be disseminated and adopted in order to reduce the risk factor of early conduct problems.

## 4. Ethics and dissemination

The study has been approved by the Swedish Ethical Review Authority (2020–06804). As the allocations consist of either a previously tested intervention or usual practice, the trial is deemed to pose minimal risk to participants. Forms for informed consent have not been used as the trial will not collect personal or sensitive information. Data will still be collected through or in conjunction with locked systems, only accessible by the research group, for interim storage. All raw data will then be stored on a secure platform (LUSEC) with access restricted to the PI and data manager.

The trial results will primarily be disseminated through two manuscripts: One manuscript will detail results regarding research questions centered on changes in primary and secondary outcomes. This manuscript will report according to CONSORT 2010 Statement (54). The other manuscript will focus on research questions regarding implementation. This study protocol is reported according to SPIRIT-guidelines (55), see Supplementary Table S2 for a SPIRIT Checklist with additional details.

## 5. Trial status

The trial has currently completed data collection. Any other major updates, amendments or revisions regarding the trial will be reported to this journal and ClinicalTrials.gov. As the trial has currently completed phases requiring participant involvement, any new amendments are not expected to require further reviewing by the Swedish Ethical Review Authority. Though should such amendments occur, they will be reported to relevant parties.

The current protocol version in use is 3.0 (dated 3 May 2023). Major changes from version 2.0 (dated 14 January 2021) to 3.0 include: Clearly specifying primary outcome and endpoint, changing from constrained to stratified randomization, reducing the number of classrooms observed by independent and blinded observers, removing old power analysis, and specifying details according to SPIRIT-guidelines. Major changes from version 1.0 (dated 16 June 2020) to version 2.0 include: removing individual-level measures.

## Ethics statement

The studies involving humans were approved by Swedish Ethical Review Authority. The studies were conducted in accordance with the local legislation and institutional requirements. Written informed consent for participation was not required from the participants or the participants’ legal guardians/next of kin because the trial does not collect personal or sensitive information.

## Author contributions

DD: Conceptualization, Funding acquisition, Methodology and Project administration, Writing – original draft, Writing – review & editing. MB: Conceptualization, Funding acquisition, Methodology and Project administration, Writing – review & editing. PA: Writing – review & editing. BH: Conceptualization, Funding acquisition, Methodology, Project administration and Supervision, Writing – review & editing.

## References

[1] Weare K Nind M. Mental health promotion and problem prevention in schools: what does the evidence say? Health Promot Int. (2011) 26:i29–69. doi: 10.1093/heapro/dar075, PMID: 22079935

[2] Schmidt M Werbrouck A Verhaeghe N Putman K Simoens S Annemans L. Universal mental health interventions for children and adolescents: a systematic review of health economic evaluations. Appl Health Econ Health Policy. (2020) 18:155–75. doi: 10.1007/s40258-019-00524-0, PMID: 31605299

[3] Albee GW Ryan K. An overview of primary prevention. J Ment Health. (1998) 7:441–9. doi: 10.1080/09638239817824

[4] Barrish HH Saunders M Wolf MM. Good behavior game: effects of individual contingencies for group consequences on disruptive behavior in a classroom. J Appl Behav Anal. (1969) 2:119–24. doi: 10.1901/jaba.1969.2-119, PMID: 16795208 PMC1311049

[5] Smith S Barajas K Ellis B Moore C McCauley S Reichow B. A Meta-analytic review of randomized controlled trials of the good behavior game. Behav Modif. (2021) 45:641–66. doi: 10.1177/0145445519878670, PMID: 31578077

[6] American Psychiatric Association. Diagnostic and statistical manual of mental disorders. Fifth ed, Text Revision (DSM-5-TR) Washington, DC: American Psychiatric Association Publishing. (2022).

[7] Barker ED Maughan B. Differentiating early-onset persistent versus childhood-limited conduct problem youth. Am J Psychiatr. (2009) 166:900–8. doi: 10.1176/appi.ajp.2009.08121770, PMID: 19570930

[8] Odgers CL Moffitt TE Broadbent JM Dickson N Hancox RJ Harrington H . Female and male antisocial trajectories: from childhood origins to adult outcomes. Dev Psychopathol. (2008) 20:673–716. doi: 10.1017/S0954579408000333, PMID: 18423100

[9] Sentse M Kretschmer T de Haan A Prinzie P. Conduct problem trajectories between age 4 and 17 and their association with behavioral adjustment in emerging adulthood. J Youth Adolesc. (2017) 46:1633–42. doi: 10.1007/s10964-016-0476-4, PMID: 27017600 PMC5491626

[10] Kjeldsen A Janson H Stoolmiller M Torgersen L Mathiesen KS. Externalising behaviour from infancy to mid-adolescence: latent profiles and early predictors. J Appl Dev Psychol. (2014) 35:25–34. doi: 10.1016/j.appdev.2013.11.003

[11] Fanti KA Hellfeldt K Colins OF Meehan A Andershed A-K Andershed H. Worried, sad, and breaking rules? Understanding the developmental interrelations among symptoms of anxiety, depression, and conduct problems during early childhood. J Crim Just. (2019) 62:23–8. doi: 10.1016/j.jcrimjus.2018.09.006

[12] Suldo SM Gormley MJ DuPaul GJ Anderson-Butcher D. The impact of school mental health on student and school-level academic outcomes: current status of the research and future directions. Sch Ment Heal. (2013) 6:84–98. doi: 10.1007/s12310-013-9116-2

[13] Weeks M Ploubidis GB Cairney J Wild TC Naicker K Colman I. Developmental pathways linking childhood and adolescent internalizing, externalizing, academic competence, and adolescent depression. J Adolesc. (2016) 51:30–40. doi: 10.1016/j.adolescence.2016.05.009 27288965

[14] Bevilacqua L Hale D Barker ED Viner R. Conduct problems trajectories and psychosocial outcomes: a systematic review and meta-analysis. Eur Child Adolesc Psychiatry. (2018) 27:1239–60. doi: 10.1007/s00787-017-1053-4, PMID: 28983792

[15] Hammerton G Edwards AC Mahedy L Murray J Maughan B Kendler KS . Externalising pathways to alcohol-related problems in emerging adulthood. J Child Psychol Psychiatry. (2020) 61:721–31. doi: 10.1111/jcpp.13167, PMID: 31769047 PMC7242151

[16] Kellam SG Brown CH Poduska JM Ialongo NS Wang W Toyinbo P . Effects of a universal classroom behavior management program in first and second grades on young adult behavioral, psychiatric, and social outcomes. Drug Alcohol Depend. (2008) 95:S5–S28. doi: 10.1016/j.drugalcdep.2008.01.004, PMID: 18343607 PMC2512256

[17] Kellam SG Wang W Mackenzie AC Brown CH Ompad DC Or F . The impact of the good behavior game, a universal classroom-based preventive intervention in first and second grades, on high-risk sexual behaviors and drug abuse and dependence disorders into young adulthood. Prev Sci. (2014) 15:6–18. doi: 10.1007/s11121-012-0296-z, PMID: 23070695 PMC3808514

[18] Petras H Kellam SG Brown CH Muthen BO Ialongo NS Poduska JM. Developmental epidemiological courses leading to antisocial personality disorder and violent and criminal behavior: effects by young adulthood of a universal preventive intervention in first- and second-grade classrooms. Drug Alcohol Depend. (2008) 95:S45–59. doi: 10.1016/j.drugalcdep.2007.10.015, PMID: 18243581 PMC2706504

[19] Wilcox HC Kellam SG Brown CH Poduska JM Ialongo NS Wang W . The impact of two universal randomized first- and second-grade classroom interventions on young adult suicide ideation and attempts. Drug Alcohol Depend. (2008) 95:S60–73. doi: 10.1016/j.drugalcdep.2008.01.005, PMID: 18329189 PMC2637412

[20] Nolan JD Houlihan D Wanzek M Jenson WR. The good behavior game: a classroom-behavior intervention effective across cultures. Sch Psychol Int. (2013) 35:191–205. doi: 10.1177/0143034312471473

[21] Ashworth E Humphrey N Hennessey A. Game over? No Main or subgroup effects of the good behavior game in a randomized trial in English primary schools. J Res Educ Effect. (2020) 13:298–321. doi: 10.1080/19345747.2019.1689592

[22] Sundell K Beelmann A Hasson H von Thiele SU. Novel programs, international adoptions, or contextual adaptations? Meta-analytical results from German and Swedish intervention research. J Clin Child Adolesc Psychol. (2016) 45:784–96. doi: 10.1080/15374416.2015.1020540 25864716

[23] Domitrovich CE Bradshaw CP Poduska JM Hoagwood K Buckley JA Olin S . Maximizing the implementation quality of evidence-based preventive interventions in schools: a conceptual framework. Adv School Ment Health Promot. (2008) 1:6–28. doi: 10.1080/1754730X.2008.9715730, PMID: 27182282 PMC4865398

[24] Sundell K Ferrer-Wreder L Fraser MW. Going global: a model for evaluating empirically supported family-based interventions in new contexts. Eval Health Prof. (2014) 37:203–30. doi: 10.1177/0163278712469813 23291390

[25] Durlak JA DuPre EP. Implementation matters: a review of research on the influence of implementation on program outcomes and the factors affecting implementation. Am J Community Psychol. (2008) 41:327–50. doi: 10.1007/s10464-008-9165-0, PMID: 18322790

[26] Humphrey N Panayiotou M Hennessey A Ashworth E. Treatment effect modifiers in a randomized trial of the good behavior game during middle childhood. J Consult Clin Psychol. (2021) 89:668–81. doi: 10.1037/ccp0000673, PMID: 34472894

[27] Ulla T Poom-Valickis K. Program support matters: a systematic review on teacher – and school related contextual factors facilitating the implementation of social-emotional learning programs. Frontiers. Education. (2023) 7:7. doi: 10.3389/feduc.2022.965538

[28] Moore JE Bumbarger BK Cooper BR. Examining adaptations of evidence-based programs in natural contexts. J Prim Prev. (2013) 34:147–61. doi: 10.1007/s10935-013-0303-6, PMID: 23605294

[29] Loudon K Treweek S Sullivan F Donnan P Thorpe KE Zwarenstein M. The PRECIS-2 tool: designing trials that are fit for purpose. BMJ. (2015) 350:h 2147. doi: 10.1136/bmj.h2147, PMID: 25956159

[30] Hawkins JD. Preventing crime and violence through communities that care. Eur J Crim Policy Res. (1999) 7:443–58. doi: 10.1023/A:1008769321118

[31] Tingstrom DH Sterling-Turner HE Wilczynski SM. The good behavior game: 1969-2002. Behav Modif. (2006) 30:225–53. doi: 10.1177/0145445503261165, PMID: 16464846

[32] Donaldson JM Wiskow KM. The good behavior game. In: Preventing Crime and Violence. Advances in Prevention Science. eds. Teasdale B. Bradley MS (2017) 229–41.

[33] Leflot G van Lier PA Onghena P Colpin H. The role of teacher behavior management in the development of disruptive behaviors: an intervention study with the good behavior game. J Abnorm Child Psychol. (2010) 38:869–82. doi: 10.1007/s10802-010-9411-4, PMID: 20373016

[34] van Lier PA Muthen BO van der Sar RM Crijnen AA. Preventing disruptive behavior in elementary schoolchildren: impact of a universal classroom-based intervention. J Consult Clin Psychol. (2004) 72:467–78. doi: 10.1037/0022-006X.72.3.467, PMID: 15279530

[35] Dijkman MAM Harting J van der Wal MF. Adoption of the good behaviour game: an evidence-based intervention for the prevention of behaviour problems. Health Educ J. (2014) 74:168–82. doi: 10.1177/0017896914522234

[36] Koutakis N. Good Behavior Game i Sverige: En utvärdering av Höjaspelet på Höjaskolan i Malmö. Malmö: Malmö, Sweden. (2017).

[37] Kjøbli J Sørlie MA. School outcomes of a community-wide intervention model aimed at preventing problem behavior. Scand J Psychol. (2008) 49:365–75. doi: 10.1111/j.1467-9450.2008.00648.x, PMID: 18466191

[38] Sørlie MA Ogden T. Immediate impacts of PALS: a school-wide multi-level programme targeting behaviour problems in elementary school. Scand J Educ Res. (2007) 51:471–92. doi: 10.1080/00313830701576581

[39] Sørlie M-A Ogden T. School-wide positive behavior support–Norway: impacts on problem behavior and classroom climate. Int J School Educ Psychol. (2015) 3:202–17. doi: 10.1080/21683603.2015.1060912

[40] Goddard R. A theoretical and empirical analysis of the measurement of collective efficacy the development of a short form. Educ Psychol Meas. (2002) 62:97–110. doi: 10.1177/0013164402062001007

[41] Sørlie M-A Ogden T Olseth AR. Examining teacher outcomes of the school-wide positive behavior support model in Norway. SAGE Open. (2016) 6:215824401665191. doi: 10.1177/2158244016651914

[42] Robinson CH Damschroder LJ. A pragmatic context assessment tool (pCAT): using a think aloud method to develop an assessment of contextual barriers to change. Implement Sci Commun. (2023) 4:3. doi: 10.1186/s43058-022-00380-5, PMID: 36631914 PMC9835384

[43] Cataldo MF Risley TR. Development of a standardized measure of classroom participation. American Psychological Association; Montreal, Canada: U.S. National Institute of Education (1973). 1–12.

[44] Conduct Problems Prevention Research Group. Initial impact of the fast track prevention trial for conduct problems: II. Classroom Effects J Consult Clin Psychol. (1999) 67:648–57. doi: 10.1037/0022-006X.67.5.648, PMID: 10535231 PMC2761630

[45] McNeish D Stapleton LM Silverman RD. On the unnecessary ubiquity of hierarchical linear modeling. Psychol Methods. (2017) 22:114–40. doi: 10.1037/met0000078, PMID: 27149401

[46] Smid SC McNeish D Miočević M van de Schoot R. Bayesian versus frequentist estimation for structural equation models in small sample contexts: a systematic review. Struct Equ Model Multidiscip J. (2019) 27:131–61. doi: 10.1080/10705511.2019.1577140

[47] Depaoli S Winter SD Visser M. The importance of prior sensitivity analysis in Bayesian statistics: demonstrations using an interactive shiny app. Front Psychol. (2020) 11:608045. doi: 10.3389/fpsyg.2020.608045, PMID: 33324306 PMC7721677

[48] Brown CH Curran G Palinkas LA Aarons GA Wells KB Jones L . An overview of research and evaluation designs for dissemination and implementation. Annu Rev Public Health. (2017) 38:1–22. doi: 10.1146/annurev-publhealth-031816-044215, PMID: 28384085 PMC5384265

[49] Lane-Fall MB Curran GM Beidas RS. Scoping implementation science for the beginner: locating yourself on the "subway line" of translational research. BMC Med Res Methodol. (2019) 19:133. doi: 10.1186/s12874-019-0783-z, PMID: 31253099 PMC6599376

[50] Glasgow RE Harden SM Gaglio B Rabin B Smith ML Porter GC . RE-AIM planning and evaluation framework: adapting to new science and practice with a 20-year review. Front Public Health. (2019) 7:64. doi: 10.3389/fpubh.2019.00064, PMID: 30984733 PMC6450067

[51] Flower A McKenna JW Bunuan RL Muething CS Vega R. Effects of the good behavior game on challenging behaviors in school settings. Rev Educ Res. (2014) 84:546–71. doi: 10.3102/0034654314536781

[52] Kazdin AE. Treatment as usual and routine care in research and clinical practice. Clin Psychol Rev. (2015) 42:168–78. doi: 10.1016/j.cpr.2015.08.006 26431668

[53] Humphrey N Hennessey A Ashworth E Frearson K Black L Peterson K . Good behavior game: evaluation report and executive summary. London, England: Education Endownment Foundation (2018).

[54] Campbell MK Piaggio G Elbourne DR Altman DG. Consort 2010 statement: extension to cluster randomised trials. BMJ. (2012) 345:e5661-e. doi: 10.1136/bmj.e5661, PMID: 22951546

[55] Chan A-W Tetzlaff JM Altman DG Laupacis A Gøtzsche PC Krleža-Jerić K . SPIRIT 2013 statement: defining standard protocol items for clinical trials. Ann Intern Med. (2013) 158:200–7. doi: 10.7326/0003-4819-158-3-201302050-00583, PMID: 23295957 PMC5114123

