# Supplementary material for: Good behavior game – study protocol for a randomized controlled trial of a preventive behavior management program in a Swedish school context
Source: Front Psychiatry. 2023 Oct 5;14:1256714. doi: 10.3389/fpsyt.2023.1256714 (PMC10585039; doi:10.3389/fpsyt.2023.1256714)
Supplement: Supplementary file 1 [file Table_1.docx]

Supplementary Material

Good behavior game – study protocol for a randomized controlled trial of a preventive behavior management program in a Swedish school context

Dariush Djamnezhad*, Martin Bergström, Per Andrén and Björn Hofvander

*** Correspondence:** Corresponding Author: dariush.djamnezhad@med.lu.se

# Supplementary Table 1: PRECIS-2 table

This supplementary table further specifies the study’s direction as a pragmatic trial using the PRECIS-2 tool (1).

|  | **Domain** | **Score** | **Rationale** |
| --- | --- | --- | --- |
| **1** | **Eligibility Criteria** | 4 | There are no additional restrictions in eligibility in the trial compared to usual conditions. However, in accordance with recommended procedures from the preventive framework Communities that Care (CTC), usual practice may require schools to be in areas with measured risk and protective factors that match intended targets for Good Behavior Game (GBG). The trial does not impose this restriction. |
| **2** | **Recruitment Path** | 4 | Recruitment follows usual procedures at first when recruiting within CTC pilot areas. The trial has additional recruitment after this phase were the trial and corresponding intervention is open to schools outside CTC pilot areas to a greater extent compared to usual procedures. |
| **3** | **Setting** | 5 | The intervention is designed for regular Swedish primary schools which is also the target population for the trial. The trial is also conducted in the same municipality for which the intervention is developed in and primarily intended for. |
| **4** | **Organization intervention** | 5 | Intervention support and training is conducted by usual staff with no additional resources for the trial in the intervention arm. Schools in the control group do not have specific access to these trainers but are not restricted in using other resources available to them (e.g., other support provided by the municipality or purchased from external providers). |
| **5** | **Flex of experimental intervention – Delivery** | 5 | No restrictions. Certified GBG-trainers are asked to provide training to schools with their usual procedures. |
| **6** | **Flex of experimental intervention – Adherence** | 5 | No restrictions are imposed by the research group. Trainers monitor and feedback teacher adherence according to their usual procedures. |
| **7** | **Follow up** | 4 | Data collection is designed to have as little impact as possible. Teacher surveys are only requested at three time points during the school year, with each survey expected to take roughly 30 minutes to complete. Observations don’t require additional effort besides booking. |
| **8** | **Primary outcome** | 3 | The outcome is relevant to schools but is not their primary goal. The outcome is not assessed in usual practice, although similar data may be collected using other instruments. |
| **9** | **Primary analysis** | 5 | The primary analysis is based on intention-to-treat regardless of intervention delivery. |

1. Loudon K, Treweek S, Sullivan F, Donnan P, Thorpe KE, Zwarenstein M. The PRECIS-2 tool: designing trials that are fit for purpose. BMJ. 2015;350:h2147.
